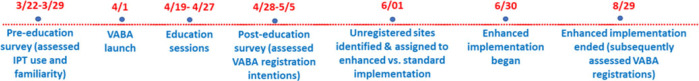# Increasing Registration for a VA Multidrug-Resistant Organism Alert Tool

**DOI:** 10.1017/ash.2023.441

**Published:** 2023-09-29

**Authors:** Cara Ray, Cassie Cunningham Goedken, Ashley Hughes, Katie Suda, Marylou Guihan, Geneva Wilson, Natalie Hicks, Martin Evans, Makoto Jones, Christopher Pfeiffer, Margaret Fitzpatrick, Stacey Klutts, Charlesnika Evans

## Abstract

**Objectives:** To address the importation of multi-drug-resistant organisms (MDROs) when a colonized or infected patient is transferred from another VA facility, the Veterans Health Administration (VHA) launched the Inpatient Pathogen Tracker (IPT) in 2020. IPT tracks MDRO-infected/colonized patients and alerts MDRO Program Coordinators (MPCs) and Infection Preventionists (IPs) when such patients are admitted to their facility to facilitate rapid identification and isolation of infected/colonized patients. IPT usage has been low during initial rollout (32.5%). The VHA and the CARRIAGE QUERI Program developed targeted implementation strategies to increase utilization of IPT’s second iteration, VA Bug Alert (VABA). **Methods:** Familiarity with IPT was assessed via pre-education survey (3/2022). All sites received standard VABA implementation including: 1) adaptation of VABA features based on end-user feedback (completed 4/2022), 2) development and delivery of an educational module regarding the revised tool (completed 4/2022), and 3) internal facilitation from the VHA MDRO Program Office (ongoing) (see Figure for all key timepoints). Intent to register for VABA was assessed via post-education survey (4-5/2022). Sites (125 eligible) not registered for VABA by 6/1/2022 were randomly assigned to receive one of two conditions from 6/2022–8/2022: continued standard implementation alone or enhanced implementation. Enhanced implementation added the following to standard implementation: 1) audit and feedback reports and 2) external facilitation, including interviews and education about VABA. We compared the number of sites with ≥1 MPC/IP registered for VABA to-date between implementation conditions. **Results:** *Pre-education survey.* 168 MPC/IPs across 117 sites responded (94% of eligible sites). Among respondents, 25% had used IPT, 35.1% were familiar with but had not used IPT, and 39.9% were unfamiliar with IPT. *Post-education survey.* 93 MPC/IPs across 80 sites responded (59% of eligible sites). Of these, 81.7% said they planned to register for VABA, 4.3% said they would not register, and 14.0% said they were unsure. *Post-6/1/2022 Registrations.* By 6/1/2022, 71% of sites had ≥1 registered VABA user. Of the 28 unregistered sites eligible for enhanced implementation, thirteen were assigned to receive enhanced implementation, and fifteen were assigned to receive continued standard implementation. Eight sites in the enhanced implementation condition (61.5%) registered for VABA. Seven standard-implementation-only sites (46.7%) registered. The number of registered sites did not significantly differ by implementation condition (Fisher’s exact *p*=0.476). **Conclusions:** Standard and enhanced implementation were equally effective at encouraging VABA registration, suggesting that allocating resources to enhanced implementation may not be necessary.

**Disclosures:** None.

**Figure:**